# Simulating impacts of rapid forest loss on population size, connectivity and genetic diversity of Sunda clouded leopards (*Neofelis diardi*) in Borneo

**DOI:** 10.1371/journal.pone.0196974

**Published:** 2018-09-12

**Authors:** Ewan A. Macdonald, Samuel A. Cushman, Erin L. Landguth, Andrew J. Hearn, Yadvinder Malhi, David W. Macdonald

**Affiliations:** 1 Environmental Change Institute, School of Geography and the Environment, University of Oxford, Oxford, United Kingdom; 2 Wildlife Conservation Research Unit, Zoology Department, University of Oxford, The Recanati-Kaplan Centre, Tubney, Abingdon, United Kingdom; 3 Rocky Mountain Research Station, United States Forest Service, Flagstaff, Arizona, United States of America; 4 Division of Biological Sciences, University of Montana, Missoula, Montana, United States of America; Universita degli Studi di Napoli Federico II, ITALY

## Abstract

Habitat loss is the greatest threat to biodiversity in Borneo, and to anticipate and combat its effects it is important to predict the pattern of loss and its consequences. Borneo is a region of extremely high biodiversity from which forest is being lost faster than in any other. The little-known Sunda clouded leopard (*Neofelis diardi*) is the top predator in Borneo and is likely to depend critically on habitat connectivity that is currently being rapidly lost to deforestation. We modeled the effects of landscape fragmentation on population size, genetic diversity and population connectivity for the Sunda clouded leopard across the entirety of Borneo. We modelled the impacts of land use change between the years 2000, 2010 and projected forwards to 2020. We found substantial reductions across all metrics between 2000 and 2010: the proportion of landscape connected by dispersal fell by approximately 12.5% and the largest patch size declined by around 15.1%, leading to a predicted 11.4% decline in clouded leopard numbers. We also predict that these trends will accelerate greatly towards 2020, with the percentage of the landscape being connected by dispersal falling by about 57.8%, the largest patch size falling by around 62.8% and the predicted clouded leopard population falling by 62.5% between 2010 and 2020. We predicted that these large declines in clouded leopard population size and connectivity will also substantially reduce the genetic diversity of the remaining clouded leopard population.

## Introduction

To assess the impacts of habitat loss and fragmentation on populations and to develop effective conservation responses it is necessary to quantify the impacts of landscape change on population extent, density and connectivity [1, 2]. However, it is difficult to predict population connectivity reliably [2, 3] given that it is scale, species and system dependent [4]. Specifically, population connectivity is the result of the combined effects of the distribution and density of the population, composition and configuration of the landscape, and species-specific dispersal characteristics (including effects of different landscape features on movement, and the effects of sex and age differences in dispersal), and how these combine to shape the dispersal kernel [5]. In most populations there is substantial uncertainty about species distributions and densities [4], how different landscape features affect movement [6], and limited understanding of species dispersal abilities [7]. These compounding uncertainties limit the confidence that can be placed in the results of many connectivity analyses.

In addition to major gaps in knowledge about the ecology of organisms and their responses to landscape conditions, there are significant technical challenges in producing fine-scale, spatially explicit predictions of population connectivity across large areas. Traditional approaches to connectivity modelling have been based on mapping least cost paths or corridors between a few “core” habitats or protected areas [8–10], which limits the generality and scope of inferences [5]. For many questions it is better to adopt spatially synoptic modelling in which connectivity is assessed continuously across space, from all locations to all others [11–13]. The challenge arises because the tools commonly available for least cost path and corridor analysis have not enabled such synoptic analysis [14]; however, recent technical advances and improved computational power combine to enable scientists to predict more robustly the effects of landscape structure and fragmentation on population connectivity in a spatially synoptic framework. For example, resistant kernel [15–17] and factorial least-cost path approaches [18–20], coupled with landscape pattern analysis [21–23], provide a framework within which to predict the location of core habitats, barriers, and movement corridors for a range of dispersal abilities across vast landscapes [5, 24–26].

Tropical forests are among the most biodiverse habitats on earth, and provide a myriad of environmental services [27–30]. However, these habitats are being lost at a devastating rate, with estimates suggesting that approximately 230 million ha of forest were destroyed globally between 2000–2012 [31]. Of all countries, it was estimated that Indonesia had experienced the largest increase in the rate of forest loss [31], and that Borneo lost 30.2% of its forest area between 1973 and 2010 [32].

The Sunda clouded leopard (*Neofelis diardi*) is the apex carnivore in Borneo, an ecologically important role that adds urgency to remedying the currently minimal knowledge of its ecology, which is based largely on observations in captivity [33], anecdotal reports [34, 35] and a few recent field studies [36, 37]. However, despite this lack of ecological knowledge, the clouded leopard is charismatic and attractive to a global public [38] and has the potential to act as a flagship for wider biodiversity conservation on Borneo.

Brodie, Giordano [9] investigated clouded leopard corridors as part of a multi-species conservation approach for Sarawak. However, they employed least cost path and circuit theory to identify the optimal corridors between chosen pairs of protected areas—a completely different methodology to the spatially synoptic approaches we adopt (resistant kernel and factorial least cost path analysis; Cushman, Lewis [14]). Specifically, we address uncertainties in landscape resistance by convening a panel of experts on the species and combining their opinions. The use of expert opinion is increasing in conservation, in part due to the increasing urgency of conservation decision-making, and in particular in situations where empirically derived field data are limited [39]. We address uncertainties in dispersal ability by modelling across two levels of dispersal (S1 File), and address uncertainty in species distribution by estimating distribution and density across space as proportional to local habitat suitability as defined by the expert panel we convened. Finally, we use spatially synoptic approaches to evaluate patterns and changes in Borneo-wide connectivity for clouded leopards.

## Methods

The analysis encompasses the entire island of Borneo, located in South East Asia and including territories governed by three countries (Brunei, Indonesia and Malaysia). The extent is just over 730,000km^2^.

We produced resistance maps for the years 2000 and 2010 for the island of Borneo based on the application of expert opinion regarding the relative habitat suitability for clouded leopards of different land cover classes. The land cover classes were taken from existing land cover maps produced for 2000 and 2010 [40] and included: water, peat swamp forest, lowland forest, lower montane forest, plantations/regrowth, lowland mosaic, montane mosaic, lowland open, montane open, urban and large scale plantation.

### Expert elicitation

Experts are commonly defined as individuals who hold information about a given topic and are deferred to in its interpretation [41]. This knowledge can be acquired in a variety of ways including training, research, skills and personal experience [42]. We convened a panel of 13 experts selected on the basis of their direct involvement in field research relating to the ecology and conservation of clouded leopards.

Group approaches to expert elicitation can result in dilution of the full diversity of opinions through the dominance of one or more members of the group, polarization among subsets of members, and groupthink [41]. Panel members were therefore contacted individually and asked to provide a relative habitat suitability estimate for each land cover class. Respondents were provided with a short questionnaire that included some brief background questions regarding the duration, type and geographic location of their experience. They were then presented with a map of land uses in Borneo, along with a list of land use classes (the map, as well as the full descriptions for each class were taken from Miettinen, Shi [43], the descriptions are reproduced in Table 1). For each land use class participants were asked (i) whether they had any direct experience working in that habitat, (ii) to estimate the population density of *N*. *diardi* per 100km^2^ in that land use class, (iii) the relative habitat suitability of that land use class for *N*. *diardi* on an interval scale of 1–5 with 5 representing the best quality habitat and 1 representing the lowest quality habitat, (iv) for any additional information that they thought might be relevant including the sources drawn upon to produce the estimates. Respondents were asked to provide answers only for those land use classes for which they felt comfortable and were invited to contact the authors in the event that they felt any elements of the survey were unclear. Additionally a pilot version of the survey was sent to a subset of respondents for comment prior to the deployment of the final survey.

**Table 1:** Table of expert derived habitat suitability scores. The experts showed a high level of agreement in rating habitat suitability with ICC_(3,1)_: 0.66 and ICC_(3,k)_: 0.96. Descriptions of land use classes from [43].

Description	Number of responses	Min score	Max score	Standard deviation	Mean score
Water In addition to natural waterbodies, this class also includes, for example, large-scale fisheries and prawn farming areas.	9	1	2	0.314	1.111
Mangrove Mangrove area masks were created using visual image interpretation. Pixels classified as forest within these areas were labeled as mangrove.	8	1	3	0.696	1.625
Peatswamp forest Forest growing on peat soil. The term ‘forest’ refers to all forests that could not be distinguished from primary forest in visual image interpretation. Therefore, this class may include also selectively logged forests and secondary forests that have reached structural characteristics (height, canopy structure, etc.) similar to primary forest.	7	2	5	1.161	3.286
Lowland forest Forest growing on mineral soil in elevation up to 750 m above sea level.	12	4	5	0.500	4.500
Lower montane forest Forest growing on mineral soil in elevation above 750 m, up to 1500 m above sea level.	12	2	5	1.010	4.250
Upper montane forest Forest growing on mineral soil in elevation above 1500 m above sea level.	10	1	5	1.077	3.200
Plantations / regrowth Plantations and natural regrowth. This class includes areas from large-scale industrial plantations and small-holder plantations to dense shrublands and young secondary forests.	9	1	3	0.667	2.000
Lowland mosaic Sub-pixel size (250 x 250 m) mosaic of closed canopy vegetation and open areas in elevation up to 750 m above sea level. Typically consists of small plantations, agricultural fields, urban areas, patches of forest and secondary forest. Note that sparse/patchy shrub vegetation, most notably in peatland areas, falls into this class.	8	2	3	0.331	2.125
Montane mosaic Same as lowland mosaic, but occurring in elevation above 750 m above sea level.	9	1	5	1.054	2.333
Lowland open Clearances and other open areas covered by seasonal crops, remnants of original vegetation, sparse ferns/grass or low shrub. Typically agricultural areas, areas undergoing land cover change or extremely degraded areas.	7	1	2	0.495	1.429
Montane open Same as lowland open, but occurring in elevation above 750 m above sea level. In addition, this class includes some naturally bare areas in high elevation.	9	1	2	0.497	1.444
Urban Major urban areas.	8	1	1	0.000	1.000
Large scale plantation Contiguous closed canopy palm plantations larger than 2 km^2^. Great majority of these areas are expected to be oil palm, but in some parts of the region (e.g. in Mindanao and in the coastal peatlands of Sumatra), small-holder coconut plantations cover extensive contiguous areas and are classified into this class. Note that newly established and young open canopy plantations are classified into the mosaic or open classes because of mixture of soil and vegetation reflectance.	7	1	2	0.452	1.286

**Fig 1:**
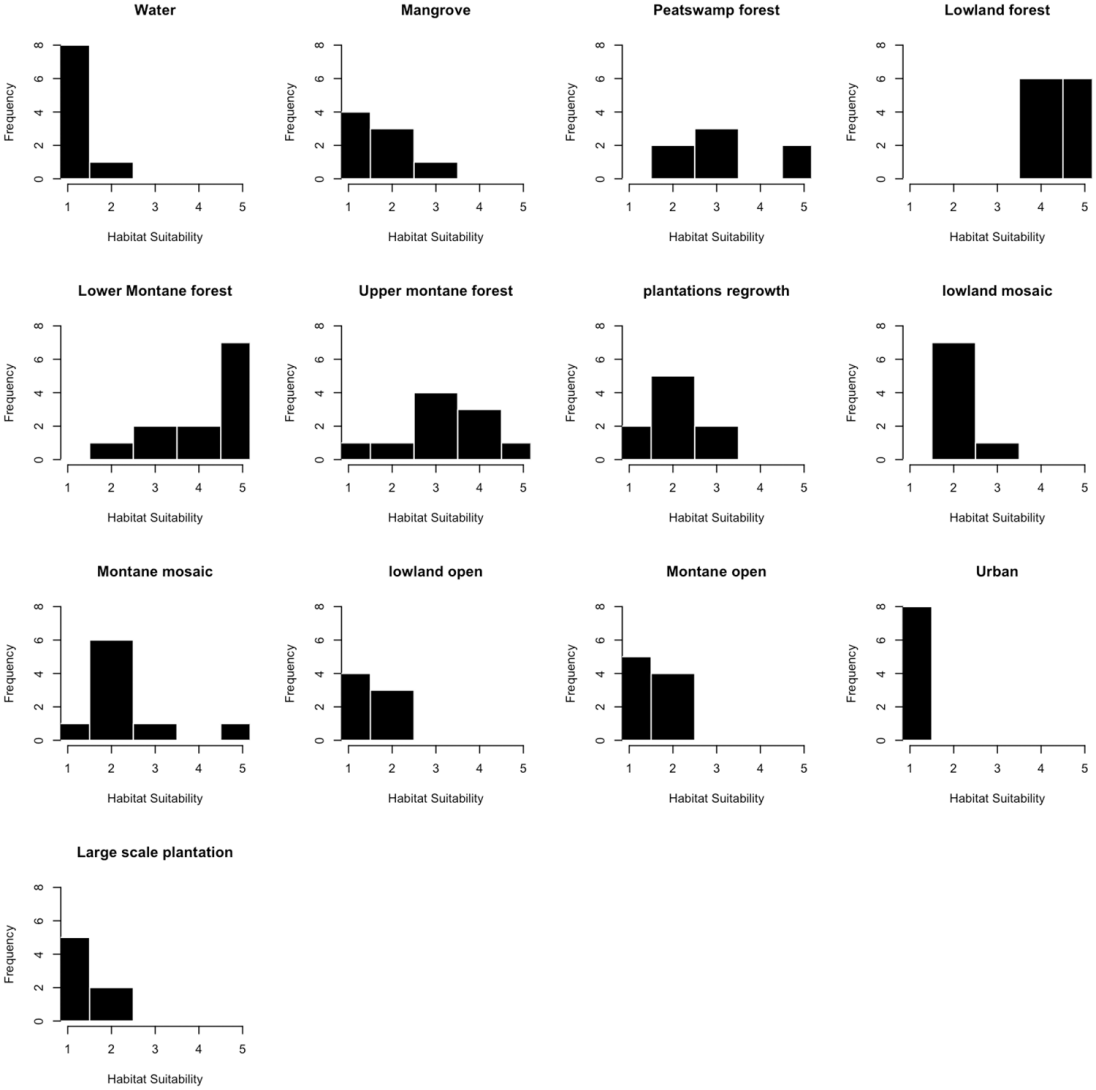
Frequency distributions of survey responses for each land use class on Borneo.

The number of respondents who felt confident to assess habitat suitability varied between each land use class ranging from a low of 7 (Peatswamp forest, Lowland open, Large scale plantation) to a high of 12 (Lowland forest, Lower Montane forest). The frequency distributions of scores allocated to each habitat class are presented in Fig 1. The Intraclass Correlation Coefficient (ICC) was high (ICC_(3,k)_: 0.96) and in excess of frequently quoted guidelines for interpretation of inter-rater agreement measures (>0.60 good, >0.75 excellent) [44, 45]. This provides confidence in the level of agreement between experts in assessing habitat suitability.

The habitat suitability score for each land use class was calculated from the mean of the habitat suitability scores provided by each respondent, this follows several similar approaches in the literature and is appropriate because responses were provided in an interval scale (Table 1)[6, 15, 46]. The mean habitat suitability scores showed a very tight relationship with the mean predicted population density for each land use class (R^2^ = 0.905, S1 File) providing further confidence in the habitat suitability scores.

### Resistance mapping in 2000 and 2010

The habitat suitability scores were translated into relative resistances by inverting and scaling from a minimum of 1 to a maximum of 100. This translation assumes that landscape resistance is directly and inversely proportional to habitat suitability, and has a minimum value of 1 in the highest quality habitat and a maximum of 100 in the lowest quality habitat. This is a very common approach that is widely used in connectivity modelling, and habitat suitability is the most common proxy adopted for landscape resistance [6, 47]. We applied this landscape resistance classification to the land cover maps for the years 2000 and 2010 [40, 43] and then resampled to a 1000m pixel size with bilinear interpolation. The resampling was done to facilitate connectivity analysis that is extremely computationally demanding and becomes intractable at fine pixel size over large areas. Cushman and Landguth [48] found that resampling resistance maps to coarser pixel size with bilinear interpolation has relatively little effect on predicted gene flow and thus is appropriate in this case to enable connectivity modeling.

### Resistance mapping for 2020

Cushman *et al*. [49] used a multi-scale random forest machine learning modelling approach [50] to predict deforestation risk across Borneo in 2020 from observed patterns of deforestation in relation to topography, landcover, human population density, and conservation status of lands between 2000 and 2010. This deforestation risk map provided the probability that a pixel that was forested in 2010 would be deforested by 2020. We used this deforestation risk map to translate the resistance map described above for 2010 to one predicted for 2020. This was done by multiplying the probability of deforestation by the resistance value for that location if it were plantations/regrowth and adding this net predicted change in resistance to the resistance value for that pixel in 2010. We did this because forest pixels lost to deforestation were most likely to transition either to plantations or regrowth.

### Defining source points for dispersers in connectivity modelling

We used the resistance layers for 2000, 2010 and 2020 to seed locations for dispersing clouded leopards. Given that the resistance surface is an inverted and scaled version of the habitat suitability map, using the resistance surface is equivalent to seeding dispersers based on estimated habitat suitability. This was done by creating a grid of random values between 0 and 1 of the same dimension and cell size as the resistance grids and then multiplying this random grid by the inverse of the resistance value for the 2000 landscape. We chose a threshold value and selected all pixels with values larger than the threshold to use source points. We selected a threshold value that would produce 2,954 source points as this plausibly approximates a conservative estimate of the effective population size of clouded leopards on Borneo [51]; however, for the purposes of this analysis the actual number is not strictly important as we report the relative changes in connectivity and gene flow rather than the absolute values. We used this same threshold for 2010 and 2020, producing 2,595 and 2,209 source points for these years, respectively.

### Connectivity modelling

#### Resistant kernel approach

The resistant kernel approach to connectivity modelling is based on all-directional least-cost dispersal from a defined set of sources [15], selected as described in the section above. The resistance maps for the three dates provide resistances for all locations in the study area, in the form of the increased cost of crossing each pixel relative to the least-cost condition. These costs are used as weights in the dispersal function, such that the expected density of dispersing individuals in a pixel is down-weighted by the cumulative cost from the source, following the least-cost route [15]. The initial expected density was set to 1 in each cell containing a source point. The model calculates the expected relative density of dispersers in each pixel around the source, given the dispersal ability of the species, the nature of the dispersal function, and the resistance of the landscape [15, 16]. We used UNICOR [17] to calculate the cumulative resistant kernel density for each resistance map at 125,000 cost units (125kcu), reflecting a maximum dispersal ability of 125km in ideal habitat, and proportionately less through higher resistance landscape conditions, and at 250,000 cost units (250kcu).

Dispersal distances are not known for clouded leopards, however, a number of studies have attempted to describe allometric relationships between maximum dispersal distances and a range of physiological and life history traits for mammals in general [52, 53]. Bowman estimates that maximum dispersal distance is equal to 40*(home range size^0.5^) and since clouded leopards are thought to have home ranges in the region of 16km^2^ (*N*. *diardi*)—40km^2^ (*N*. *nebulosa*) we therefore estimate a likely range of dispersal distances from 160 -252km [36, 52, 54, 55]. For this study we therefore selected 125km as a plausible conservative estimate of dispersal in clouded leopards, and 250kcu as a reasonable upper limit.

#### Least-cost path approach

In the second form of the connectivity modelling approach we used UNICOR [17] to produce predicted least-cost path routes from each source point to each destination point. In this factorial least cost path analysis the source points, described above, define starting and ending nodes of the least-cost paths between all pairs of individuals [3, 19]. These pair-wise least-cost paths were then combined through summation to produce maps of the density of the least-cost path network. This predicted least-cost density network was smoothed by calculating the focal mean with a 10,000m radius to better reflect local gradients of movement density [19].

### Analysis of resistant kernel maps

In their raw form the resistant kernel density maps depict the expected density of dispersing individuals. It is necessary to define a threshold kernel density for delineating connected populations. We defined connected populations as those areas greater than the 10^th^ percentile of the 2000 kernel surface and applied this threshold to all three dates (2000, 2010, 2020) to predict the extent of connected habitat across the analysis period. Given that any particular threshold is arbitrary, we decided to explore how patterns of predicted connectivity varied across various thresholds. We therefore also tested the impact of using both the 5^th^ and 20^th^ percentile of the 2000 kernel surface as thresholds in our analysis; the results of this are presented in S1 File. We reclassified the kernel density maps into binary form, with the value 1 where the kernel density probability of dispersal is greater than the percentile threshold and 0 where it is less.

We used FRAGSTATS [21] to calculate the percentage of the landscape, largest patch index, and number of patches that are predicted to be connected habitat for each year. The percentage of the landscape that is connected is the simplest metric of landscape composition, and quantifies how much of the study area is predicted to be connected habitat for each kernel map. The largest patch index [21] reports the extent, as a proportion of the size of the study area, of the largest patch of connected habitat. Lastly, we calculated the number of patches of internally connected habitat for each map, which is a simple measure of habitat subdivision or fragmentation.

### Analysis of factorial least cost path maps

In their raw form the least cost path density maps (produced for 2000, 2010, 2020) depict the location and strength of connections uniting all pairs of source points in a least-cost network. As in the kernel analysis, it is necessary to define a threshold least-cost path density for defining meaningful connections. We chose the same threshold of the 10^th^ percentile of the least cost path network value distribution for year 2000 and reclassified the least-cost path density maps into binary with the value 1 where the least cost path density is greater than the threshold and 0 where it is less (threshold values of 5^th^ and 20^th^ percentile were also tested and are presented in S1 File). We used FRAGSTATS [21] to calculate the correlation length for the least cost path density maps. Correlation length provides a measure of the average distance an organism can move within a patch before encountering the patch boundary from a random starting point [21]. The correlation length gives a global measure of the connectivity of the landscape and is a more relevant functional measure of network connectivity than more basic measures such as patch size, nearest neighbor distance, and percentage of the landscape in occupied habitat [56]. We chose to omit the other metrics since correlation length is the logical measure of the extensiveness of this network, and areal measures such as percentage of the landscape and largest patch index are less well matched to this data structure [57].

### CDPOP simulations

Simulation modelling provides explicit control over pattern-process relationships [58] and enables rigorous attribution of the causes of observed patterns of population size, genetic differentiation and diversity [59–61]. By varying functional parameters, environmental characterization, and organism attributes, simulation enables scientists to investigate hypotheses about the relative influence of different factors, their interactions, and organism characteristics, such as gradients of landscape resistance, population size or dispersal ability. This provides a means for thorough evaluation of complexes of factors that would be impossible to investigate directly in the field. In addition, simulations are particularly important in evaluating scenarios of future landscape change, such as predicted patterns of deforestation, on landscape resistance and its effects on future population genetic structure.

We used CDPOP 1.0 [20] to simulate the processes of mating and dispersal as functions of the spatial patterns of resistance across the three resistance maps corresponding to the 2000, 2010 and 2020 dates at the dispersal distances of 125kcu and 250 kcu. CDPOP is an individual based, spatially explicit, landscape genetic program that simulates birth, death, mating and dispersal of individuals in complex landscapes as probabilistic functions of movement cost among them. In each of the three landscape resistance maps, we used the same source points described above in the resistant kernel analysis as locations of individual clouded leopards. We stipulated the population to have 30 loci, with 10 alleles per locus, initially randomly assigned among individuals and a mutation rate of 0.0005, which are standard parameters widely used in landscape genetics simulation modelling We used an inverse square mating and dispersal probability function, with maximum dispersal cost-weighted distance of 125kcu, matching the dispersal ability in the resistant kernel analysis. The number of offspring was based on a Poisson probability draw with mean of 2. For each of the six resistance scenarios, we ran 10 Monte Carlo runs in CDPOP to assess stochastic variability. We simulated gene flow among these locations for 200 non-overlapping generations. Past studies have shown that this is sufficient time to ensure spatial genetic equilibrium [62]. We extracted several global measures of population genetic structure for the full study area at generation 200. These include total number of alleles in the population, observed and expected heterozygosity. We analyzed the differences in these global measures of genetic structure between 2000, 2010, and 2020.

### sGD prediction of local genetic diversity

We utilized the sGD program [59] to map local total number of alleles and observed heterozygosity. sGD uses a variable radius focal window to calculate locally centered values of a number of population genetic parameters, such as allelic richness, expected and observed heterozygosity, across spatially structured populations. We calculated the local number of alleles, observed heterozygosity and expected heterozygosity within local windows with width equal to 1/2 of the range of significant spatial genetic autocorrelation of the Mantel correlogram across the average of the 10 runs of CDPOP for each scenario [23, 63].

Several of the approaches used in this study are computationally demanding (e.g the spatially synoptic analysis of least cost paths, and the individual based population genetics models) and may not be practical for many conservation practitioners. We therefore tested whether it was possible to take a pragmatic shortcut in estimating landscape scale changes in allelic richness and heterozygosity by simply using the maps of landscape resistance, path density and kernel density. We used linear regression (using the lm library in R) to construct linear models relating these measures of genetic structure to cumulative resistant kernel value, factorial least cost path density and local average landscape resistance within a focal window of 10,000m radius to explore the relationship between these genetic attributes and landscape patterns of population connectivity.

## Results

**Fig 2:**
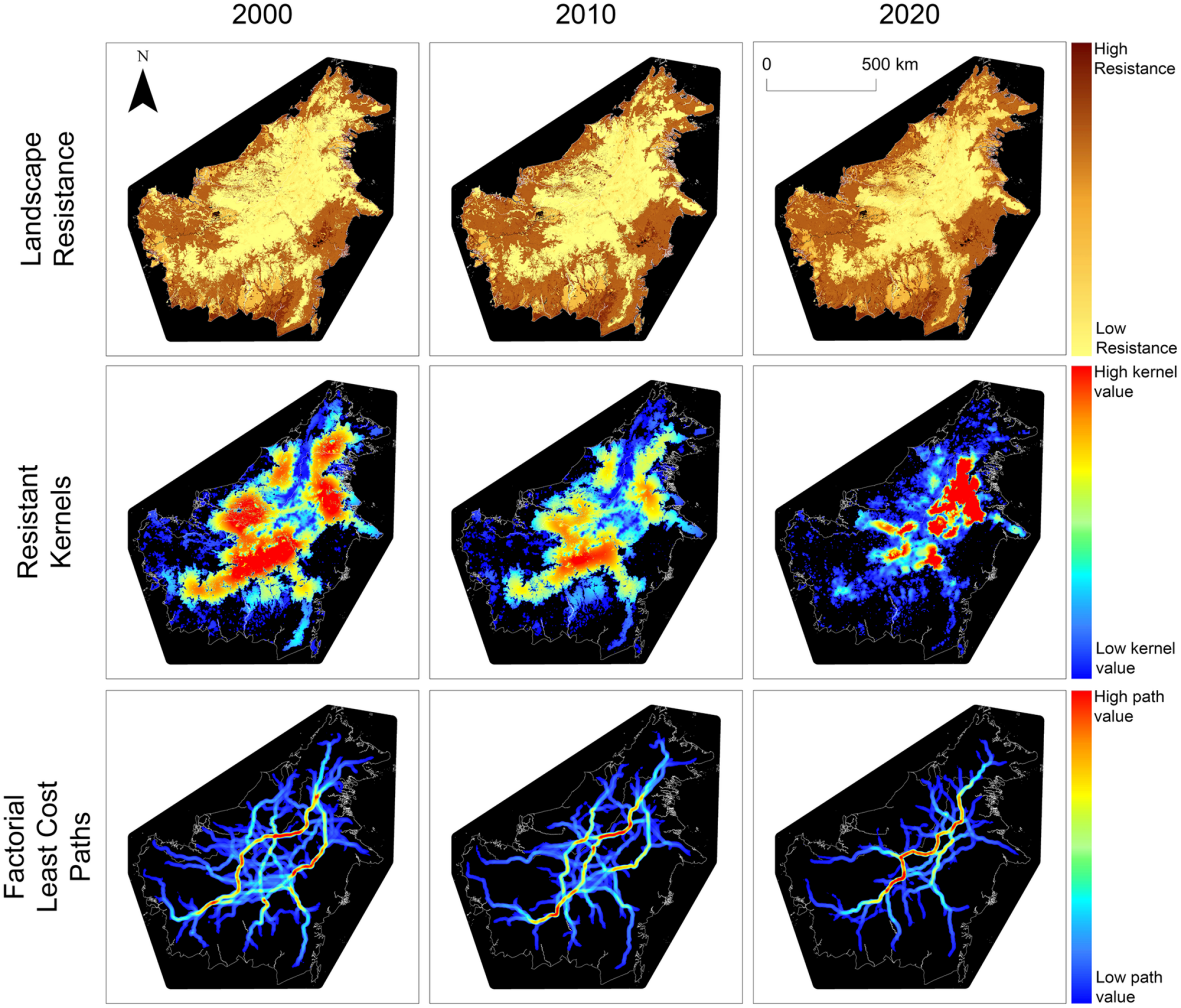
Resistance maps, cumulative resistant kernel surfaces and factorial least cost paths for 2000, 2010, 2020. Landscape resistance maps are scaled from 1 to 100, cumulative resistant kernel maps and factorial least cost path density maps are scaled from low density in blue to high density in red.

Landscape resistance change between 2000 and 2010 was driven by loss of forest cover along the edges of forested patches in Kalimantan and by a more diffuse pattern of perforation and attrition of forest patches in Sarawak and Sabah (Fig 2a and 2b). The change in landscape resistance predicted between 2010 and 2020 extended and accelerated the pattern observed between 2000 and 2010. Specifically, in Kalimantan additional forest loss was predicted primarily along the edges of existing forest patches while in Malaysian Borneo we predicted large changes in regional resistance as a result of predicted extensive and diffuse forest loss across broad areas (Fig 2c).

### Analysis of resistant kernel maps

Fig 2d, 2e and 2f shows cumulative resistant kernel density of predicted frequency of dispersal given a 125kcu dispersal threshold for the years 2000, 2010, and 2020. Change in resistant kernel density at the 250kcu threshold is shown in Figure B in S1 File.

The extent of the landscape connected by dispersal changed markedly between 2000 and 2020 (Table 2). The percentage of connected landscape declined by 12.5% between 2000 and 2010; however, this loss accelerated and there was a 57.83% decrease in the extent of the landscape connected by dispersal between 2010 and 2020 (Table 2). The relative effect of the different percentile thresholds and dispersal abilities was much less than that due to landscape change between dates (S1 File).

**Table 2:** Relative change in Fragstats metrics between 2000–2010 and 2010–2020.

	Change between 2000 and 2010	Change between 2010 to 2020
Proportion of landscape connected by dispersal	-12.46%	-57.84%
Largest patch index	-15.15%	-62.75%
Number of patches	+100%	-50%
Correlation Length	-2.5%	-9.7%

Largest patch index followed a similar pattern with the extent of the largest patch of connected habitat as percentage of the landscape falling by 15.1% between 2000 and 2010, and then 62.75% between 2010 and 2020 (Table 2). As with percentage of the landscape connected by dispersal, the change in largest patch index between years had a much greater effect than choice of dispersal threshold or percentile threshold on largest patch index (S1 File).

The number of isolated patches of internally connected habitat increased linearly, from 2 in 2000, to 4 in 2010 and to 6 in 2020 (Table 2) suggesting that large connected patches were broken up and fragmented. Selecting different kernel percentile thresholds, and dispersal distances affected the trend in the number of isolated patches, highlighting the complexity of the impacts of forest fragmentation (S1 File).

### Analysis of factorial least cost path maps

Fig 2g, 2h and 2i shows least cost path density for the years 2000, 2010, and 2020. The correlation length of the connected least cost path network decreased by 2.5% between 2000 and 2010 and by 9.7% between (Table 2). Correlation length was also sensitive to choice of percentile threshold, declining 9.2% as the threshold was moved from 5^th^ to 10^th^ percentile, and another 4.6% when moved from 10^th^ to 20^th^ percentile, for the year 2000 connectivity map (S1 File).

### CDPOP simulation results

**Fig 3:**
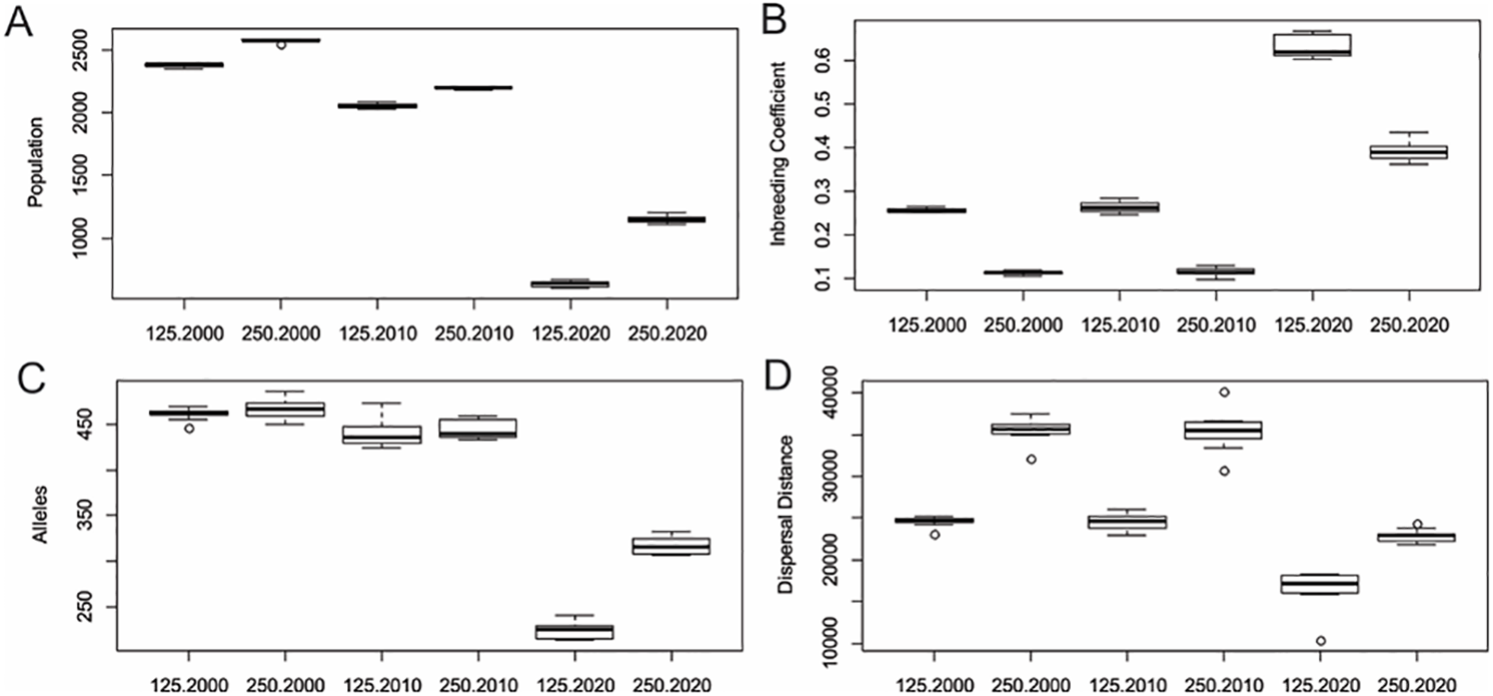
Changes in the mean population size, inbreeding coefficient, number of alleles in the population and mean dispersal distance for the years 2000, 2010, and 2020 at a dispersal distances of 125kcu and 250kcu. All values represent the equilibrium position after a simulated 200 generations of mating under each landscape condition. Each simulation was run 10 times and the points indicate the mean result and the bars indicate the standard deviation.

Over the three CDPOP scenarios (2000, 2010, 2020) there was considerable variation in simulated population size, expected heterozygosity, observed heterozygosity, average mating distance and average dispersal distance (Fig 3, Table 3). There was relatively little difference between the 125kcu and 250kcu dispersal scenarios in these population characteristics (Fig 3, Table 3). In contrast, there was a large difference between the years for each of these parameters. For example, the simulations predicted that after 200 simulated generations the population supported by the 2010 landscape was 11.4% and 14.4% smaller than that supported by the landscape in 2000, for the 125kcu and 250kcu scenarios, respectively. The simulations predicted that the clouded leopard populations in the 2020 landscape would be 62.6% and 49.8% smaller than in 2010, for the 125kcu and 250kcu scenarios, respectively.

**Table 3:** Population characteristics at generation 200 from CDPOP simulations, averages and standard deviations across 10 CDPOP runs for each scenario.

Year		Dispersal Ability	Population size	Expected Heterozygosity	Observed Heterozygosity	Inbreeding Coefficient	Alleles in the Population	Mating Distance (km)	Dispersal Distance (km)
2000	mean	125,000	2377.7	0.888	0.661	0.255	461.7	23804.4	24576.8
	stdev		16.0	0.005	0.006	0.005	6.7	578.4	599.6
2010	mean	125,000	2106.9	0.883	0.662	0.250	442.8	24998.9	25580.4
	stdev		166.9	0.006	0.045	0.049	15.7	3508.6	4153.2
2020	mean	125,000	789.0	0.792	0.341	0.575	246.5	18547.1	18300.0
	stdev		493.4	0.034	0.152	0.159	67.6	5692.8	6626.5
2000	mean	250,000	2552.2	0.887	0.777	0.124	467.6	35058.6	34280.0
	stdev		68.8	0.006	0.042	0.044	12.5	4035.2	3675.9
2010	mean	250,000	2184.9	0.879	0.767	0.127	443.7	34693.3	34394.2
	stdev		48.1	0.004	0.042	0.048	10.1	3927.6	3831.8
2020	mean	250,000	1097.3	0.824	0.485	0.413	306.4	22413.4	22501.7
	stdev		157.4	0.018	0.081	0.091	32.2	2220.0	1634.8

After 200 generations, there was relatively little predicted change in the equilibrium values for observed heterozygosity and inbreeding coefficient between the 2000 and 2010 landscapes, at either dispersal threshold. However, the simulations predicted that the 2020 landscape would lead to a 48.5% and 36.8% reduction in observed heterozygosity compared to 2010, for the 125kcu and 250kcu scenarios, respectively (Fig 3, Table 3). This corresponded to increases in simulated inbreeding coefficient of 130% and 225% respectively (Fig 3, Table 3).

### Relationships between local genetic diversity and landscape connectivity

**Fig 4:**
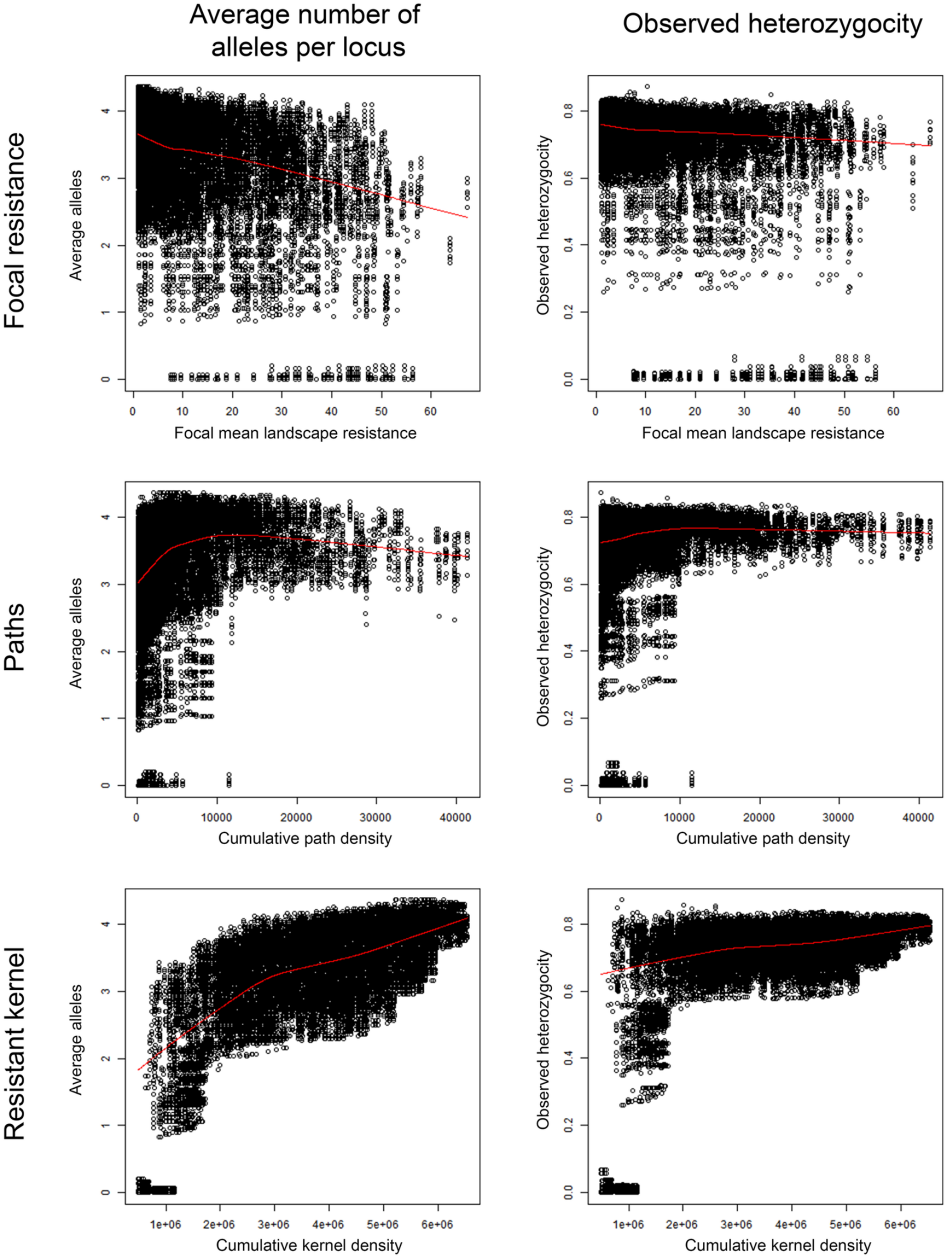
Scatterplots and fitted LOWESS splines for relationship between average number of alleles per locus (left column) and observed heterozygosity (right column) with focal mean landscape resistance within a 10km radius (top row), cumulative density of least cost paths (middle row), and cumulative resistant kernel density (bottom row).

We explored the relationships between local average number of alleles per locus (Fig 4) and local heterozygosity (Fig 4) and focal mean landscape resistance, cumulative density of least cost paths, and cumulative resistant kernel density. Visual inspection of the scatterplots suggested little relationship between allelic richness or heterozygosity and average landscape resistance within a 10km radius focal window. Cumulative density of least cost paths and cumulative resistant kernel value evinced curvilinear relationships with both allelic richness and heterozygosity in local genetic neighborhoods.

**Fig 5:**
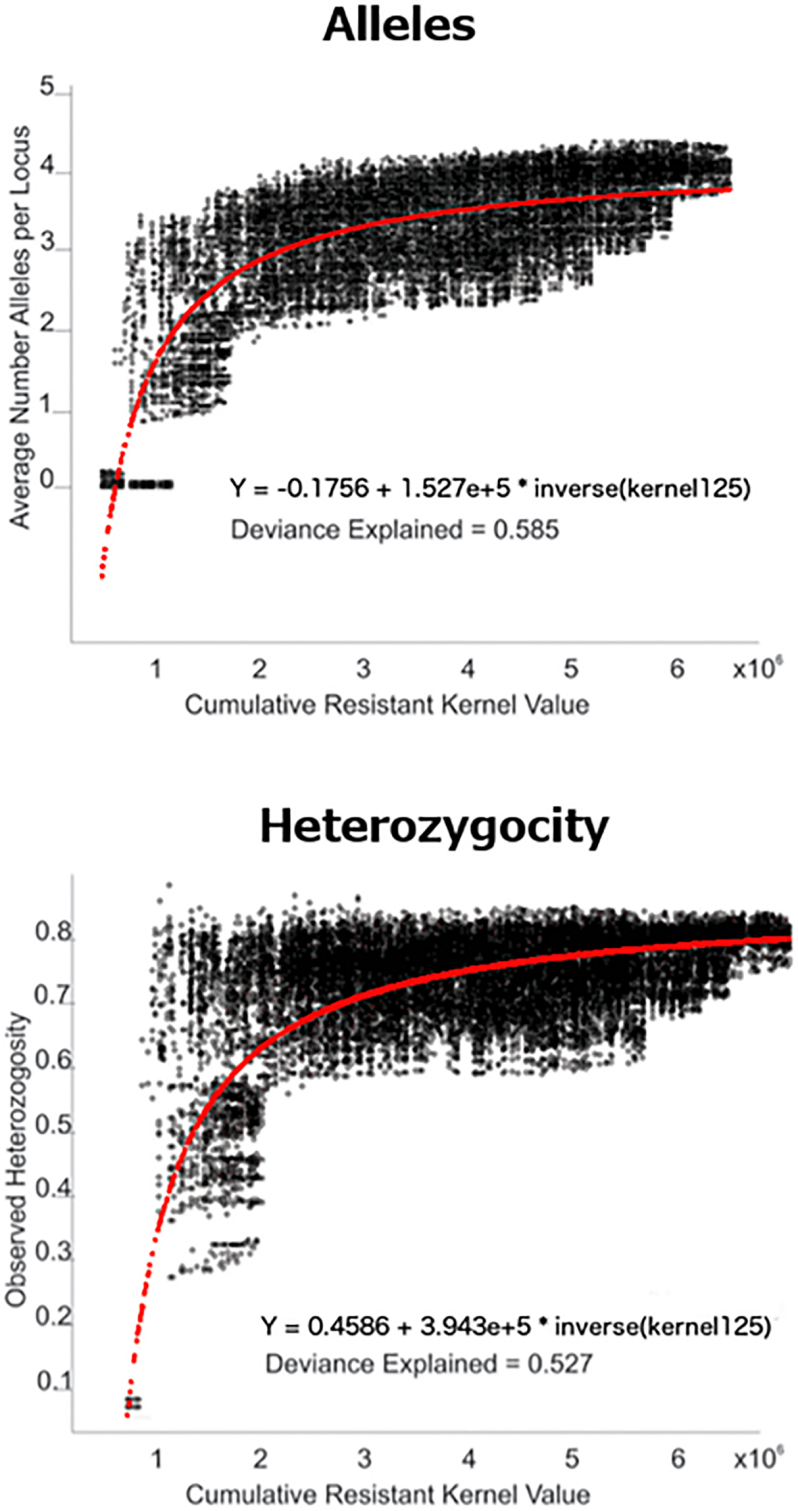
Scatterplots of (a) average number of alleles per locus and (b) observed heterozygosity in a local neighborhood of 10km radius relative to the resistant kernel value calculated with a 125kcu dispersal distance. The fitted regression equation is shown in red overlay, and the equation and deviance explained are displayed below each scatterplot.

Our regression analysis indicated that cumulative resistant kernel density was the dominant predictor, with an inverse transformation to linearize the relationship for number of alleles and the heterozygosity at 125kcu dispersal distance. We evaluated all combinations of local resistance, cumulative least cost path density and cumulative resistant kernel value for both the average alleles per locus and observed heterozygosity. We selected the single variable model with cumulative resistant kernel value as the only predictor variable, based on the observation that the single variable resistant kernel model was within 1% of the total deviance explained by the global model, and that none of the other variables’ single variable models explained more than about 15% of the deviance. This indicates that neither local landscape resistance nor cumulative least cost path density are good predictors of genetic diversity in a population. On the other hand, the resistant kernel models were remarkably good, explaining over 52% of deviance for both alleles and heterozygosity in the 125kcu dispersal scenario (Fig 5; this rose to excess of 80% of the deviance for both variables in the 250kcu dispersal distance scenario (S1 File)).

## Discussion

Ongoing rapid landscape change on Borneo is reducing the extent and increasing the fragmentation of habitat for forest dependent species, such as the clouded leopard. This reduction in habitat amount and connectivity has important repercussions for both genetic diversity and projected population sizes. Our analysis suggested rapidly accelerating reductions in the extent and connectivity of forest habitats, with large predicted decreases in clouded leopard population size, accompanied by increasing inbreeding coefficient and decreasing genetic diversity. This suggests that shrinkage and fragmentation of habitat across Borneo will not only result in smaller clouded leopard populations, but also populations that are potentially more vulnerable to demographic stochasticity and inbreeding depression as well as being less able to adapt to future environmental change [64]. These trends were robust across dispersal distances (125km and 250km) and across different percentile thresholds for clouded leopard core areas.

### Relationships between genetic diversity and landscape connectivity

This is the first study to model effects of ongoing deforestation on population decline and changes in population connectivity and genetic diversity of any species at this scale. The spatially synoptic analysis of least cost paths across Borneo in particular was extremely computationally demanding, requiring the calculation of all least cost paths between all source points (between 715,806 and 4,114,146 least cost paths for each scenario). In addition, parameterizing and running an individual based population genetics model such as CDPOP across such a vast scale and large population size represents a considerable challenge for most practitioners. Therefore, we tested whether it was possible to take a pragmatic shortcut in estimating landscape scale changes in allelic richness and heterozygosity by simply using the maps of landscape resistance, path density and kernel density that we had already computed. Our regression analysis showed that the full model (using all three predictor variables) allowed us to explain about 60% of the deviance for both allelic richness and heterozygosity. However in all cases, the single variable model (including only kernel density) was the most parsimonious solution and explained an almost identical proportion of the deviance. Future studies that are limited by time or computational power might therefore be able to rely on cumulative resistant kernel density as a proxy for changes in measures of genetic diversity.

### Conservation implications and future research

Despite a recent increase in research effort, the Sunda clouded leopard on Borneo remains elusive and one of the world’s least known felids, and this hampers the development of coherent conservation actions [51]. Nonetheless, this study highlights the severe impacts of ongoing fragmentation and loss of forest habitats on Borneo. Our analyses predicted a marked decline in both the overall area of clouded leopard core areas and a marked reduction in patch sizes with several patches being lost all together, coupled with dramatic declines in allelic richness and heterozygosity. Future conservation effort should focus on slowing the rate of land use change in Borneo, while prioritizing conservation in areas that maintain the extent of core populations and the connectivity between them. Additionally, while a small number of studies have successfully made local estimates of clouded leopard density [36, 37, 65], future research should focus on better estimates of clouded leopard abundance and density across Borneo as well as improving knowledge of the species’ basic ecology (particularly its dispersal) and how it responds to anthropogenic changes in its environment. This would improve our ability to reliably map both habitat suitability and landscape resistance. We are currently in the process of refining the models presented in this paper using empirical field data from the relatively well-studied region of Sabah, however Borneo wide initiatives are also required.

### Further implications

The implications of this study extend beyond the conservation of the Sunda clouded leopard. Firstly, while our models were parameterized specifically for clouded leopards based on expert opinion they are likely to have relevance to any other medium sized forest dependent mammals on Borneo (such as the sun bear (*Helarctos malayanus*)) that may have similar dispersal distances and habitat associations.

Macdonald, Burnham [66] have highlighted the fact that many species (for example felids and primates) face the same threats in the same locations and may benefit from many of the same solutions. Building on this notion, [67] and [38] have shown that the clouded leopard is not only regarded as charismatic by the general public but that it has strong potential to act as an Ambassador for conservation marketing campaigns. This supports the widespread approach of protecting patches or corridors that support one or several wide-ranging, often large-bodied, species on the assumption that conservation of these umbrella species will also facilitate conservation of smaller or less mobile organisms (e.g. [68, 69] but see [5, 25]). As a generalization, Beier et al. [70] argued that habitat corridors may more effectively protect regional biodiversity if they are developed to support the movement of multiple species simultaneously, because so many species are threatened by fragmentation, a proposition which underlies the analyses presented by [9] for a basket of SE Asian species including clouded leopards.

### Constraints and limitations of the approach

Some parts of our model are populated by data derived from expert opinion, and although our team includes researchers with many years of experience conducting field research on clouded leopards, maps of habitat suitability derived from expert opinion have been shown to be less reliable than empirically derived data [71–73]. In this study, the ICC suggests that survey respondents showed a high level of agreement, however inevitably the results for some land uses were more certain than others. Frequency distributions for the survey responses are shown in Fig 1, and these show that while reviewers were reasonably unanimous in their view that lowland forests represented high quality habitat, they were less consistent in their view towards peat swamp forest. The comparative uncertainty in these areas could influence the reliability of our model and highlights the need for greater research in these less understood habitat types. Reassuringly however, the estimated population density provided by the survey respondents (1.12 ind. per 100km^2^) falls within the empirically estimated range of population density for clouded leopards in the peat swamp forest of Sabangau (0.72 to 4.41 ind. per 100km^2^)[65]. For this reason it will be important to collect empirical data on population distribution, abundance, genetic diversity and gene flow to verify and improve our predictions.

Additionally, our method for estimating the landscape resistance in 2020 has inherent uncertainty. Our method involves multiplying pixel values by the probability of deforestation in each cell, and thus if we have two pixels that both have a 50% probability of deforestation by 2020, then both cells will be multiplied by 50% whereas in reality we might expect that one cell might be completely deforested and the other might remain intact. One potential solution to this inaccuracy would have been to allocate a number of cells for deforestation based on their probability of loss. Under this approach, however, we would still have no objective way of knowing which pixel would be deforested. Our approach cannot therefore be used to interpret fine scale impacts of local land use changes, although the impacts at a wider landscape scale remain robust. Nonetheless, the present urgency of guiding conservation in Borneo requires prompt action to address an unfolding biodiversity crisis. Therefore, we believe the governments of Borneo should work with conservationists, scientists and economists to utilize the best information currently available to develop proactive strategies to slow the dramatic pace of forest loss and biodiversity impacts. It was our goal in this paper to help provide some information for this effort.

### Conclusion

Deforestation is on-going and accelerating across Borneo with immense implications for biodiversity. Our analysis is the first to use robust predictions of future landscape change at such a broad scale to simulate changes in population size, connectivity, gene flow and genetic diversity. Our results suggest large recent declines in clouded leopard population size and genetic diversity and predict much larger imminent declines as deforestation continues across Borneo, particularly in Sabah and Sarawak, where the pattern of forest loss is much more damaging due to its widespread, diffuse nature. Our analyses provide plausible inferences about how recent and future landscape change may affect clouded leopard populations (and probably those of other species with similar biology) across Borneo. Additional work using extensive occurrence, genetic and movement data is needed to verify and improve our predictions. However, given the immediate and urgent need for effective conservation action in the part of the world experiencing the most rapid loss of tropical forest, we hope our analyses could be of immediate utility in identifying (1) population core areas for priority conservation, (2) linkages between these core areas, (3) relationships between habitat extent, landscape resistance, population size, dispersal ability and genetic diversity to help managers and planners anticipate the effects of a range of alternative conservation scenarios. The tools we have presented here could be of use in developing and evaluating the specific impacts of alternative scenarios on population size, connectivity and genetic diversity.

## Supporting information

S1 File Supporting information for simulating impacts of rapid forest loss on population size, connectivity and genetic diversity of Sunda clouded leopards (*Neofelis diardi*) in Borneo. **Fig A. Plot of the mean respondent scores for habitat suitability vs the mean estimated population density for clouded leopards**. The high of R^2^ = 0.905 shows high consistency in the estimates provided by the panel of experts. **Fig B. Comparison of resistant kernel maps for two potential dispersal distances**. 125kcu (as shown in Fig 2 -,A, B, C, and 250kcu, D, E, F, for years 2000 (A and D), 2010 (B and E) and 2020 (C and F). **Fig C. Change in the percentage of the landscape connected by dispersal for 2000, 2010, and 2020 across varying thresholds for selection and dispersal distance. Fig D. Change in the extent of the largest patch of connected habitat as percentage of the landscape across the 5**^**th**^**, 10**^**th**^ **and 20**^**th**^ **percentiles of the cumulative kernel surface in year 2000 for 2000, 2010, and 2020 at (a) 125kcu and (b) 250kcu dispersal thresholds. Fig E. Change in the number of isolated patches of connected habitat across the 5**^**th**^**, 10**^**th**^ **and 20**^**th**^ **percentiles of the cumulative kernel surface in year 2000 for years 2000, 2010, and 2020 at (a) 125kcu and (b) 250kcu dispersal thresholds. Fig F. Changes in the correlation length of the factorial least cost path network across three density thresholds (5**^**th**^ **percentile, 10**^**th**^ **percentile and 20**^**th**^ **percentile of the least cost path density in 2000), and across the three dates (2000, 2010, 2020). Fig G. Scatterplots and fitted LOWESS splines for relationship between average number of alleles per locus and focal mean landscape resistance within a 10km radius (column 1), cumulative density of least cost paths (column 2), and cumulative resistant kernel density (column 3), across the two dispersal distance scenarios (125kcu, row 1; 250kcu, row 2). Fig H. Scatterplots and fitted LOWESS splines for relationship between observed heterozygosity and focal mean landscape resistance within a 10km radius (column 1), cumulative density of least cost paths (column 2), and cumulative resistant kernel density (column 3), across the two dispersal distance scenarios (125kcu, row 1; 250kcu, row 2). Fig I. Scatterplots of average number of alleles per locus in a local neighborhood relative to resistant kernel value for a) the 125kcu scenario and b) the 250kcu scenario, and heterozygosity of the local neighborhood relative to resistant kernel value for c) the 125kcu scenario and d) the 250kcu scenario. The fitted regression equation is shown in red overlay, and the equation and deviance explained are displayed below each scatterplot**.
(DOCX)

S2 File Core Areas and Corridors data for PLOS—LCP and Resistance. This .zip file contains the following shapefiles:
Landscape Resistance map for the year 2000 (“Resistance_2000.tif”)Landscape Resistance map for the year 2010 (“Resistance_2010.tif”)Landscape Resistance map for the year 2020 (“Resistance_2020.tif”)Least Cost Path map for the year 2000 (“LCP_2000.tif”)Least Cost Path map for the year 2000 (“LCP_2010.tif”)Least Cost Path map for the year 2000 (“LCP_2020.tif”).
(ZIP)

S3 File Core Areas and Corridors data for PLOS—Kernels. This .zip file contains the following shapefiles:
Resistant kernel map for year 2000 with dispersal threshold of 125kcu (“kernel_2000_125kcu.tif”)Resistant kernel map for year 2010 with dispersal threshold of 125kcu (“kernel_2010_125kcu.tif”)Resistant kernel map for year 2020 with dispersal threshold of 125kcu (“kernel_2020_125kcu.tif”)Resistant kernel map for year 2000 with dispersal threshold of 250kcu (“kernel_2000_250kcu.tif”)Resistant kernel map for year 2010 with dispersal threshold of 250kcu (“kernel_2010_250kcu.tif”)Resistant kernel map for year 2020 with dispersal threshold of 250kcu (“kernel_2020_250kcu.tif”).
(ZIP)
