# Supplementary material for: Simulating impacts of rapid forest loss on population size, connectivity and genetic diversity of Sunda clouded leopards (Neofelis diardi) in Borneo
Source: PLoS One. 2018 Sep 12;13(9):e0196974. doi: 10.1371/journal.pone.0196974 (PMC6135353; doi:10.1371/journal.pone.0196974)
Supplement: S1 File — Fig A. Plot of the mean respondent scores for habitat suitability vs the mean estimated population density for clouded leopards. The high of R2 = 0.905 shows high consistency in the estimates provided by the panel of experts. Fig B. Comparison of resistant kernel maps for two potential dispersal distances. 125kcu (as shown in Fig 2 -,A, B, C, and 250kcu, D, E, F, for years 2000 (A and D), 2010 (B and E) and 2020 (C and F). Fig C. Change in the percentage of the landscape connected by dispersal for 2000, 2010, and 2020 across varying thresholds for selection and dispersal distance. Fig D. Change in the extent of the largest patch of connected habitat as percentage of the landscape across the 5th, 10th and 20th percentiles of the cumulative kernel surface in year 2000 for 2000, 2010, and 2020 at (a) 125kcu and (b) 250kcu dispersal thresholds. Fig E. Change in the number of isolated patches of connected habitat across the 5th, 10th and 20th percentiles of the cumulative kernel surface in year 2000 for years 2000, 2010, and 2020 at (a) 125kcu and (b) 250kcu dispersal thresholds. Fig F. Changes in the correlation length of the factorial least cost path network across three density thresholds (5th percentile, 10th percentile and 20th percentile of the least cost path density in 2000), and across the three dates (2000, 2010, 2020). Fig G. Scatterplots and fitted LOWESS splines for relationship between average number of alleles per locus and focal mean landscape resistance within a 10km radius (column 1), cumulative density of least cost paths (column 2), and cumulative resistant kernel density (column 3), across the two dispersal distance scenarios (125kcu, row 1; 250kcu, row 2). Fig H. Scatterplots and fitted LOWESS splines for relationship between observed heterozygosity and focal mean landscape resistance within a 10km radius (column 1), cumulative density of least cost paths (column 2), and cumulative resistant kernel density (column 3), across the two dispersal distan [file pone.0196974.s001.docx]

**Simulating impacts of rapid forest loss on population size, connectivity and genetic diversity of clouded leopards in Borneo.**

**Supplementary material**

**
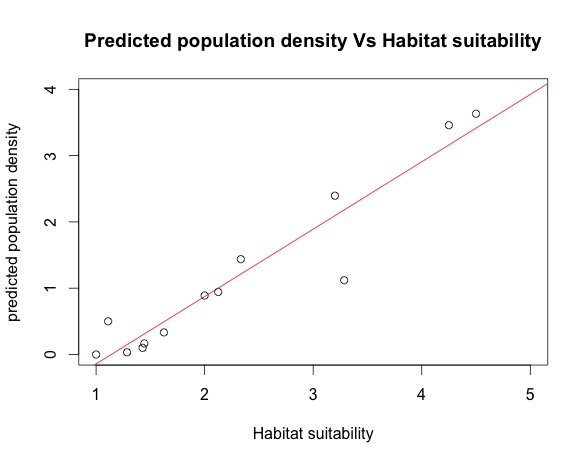
**

Figure A1. Plot of the mean respondent scores for habitat suitability vs the mean estimated population density for clouded leopards. The high of R^2^= 0.905 shows high consistency in the estimates provided by the panel of experts.

In the main body of this paper we state that “*Data on maximum dispersal distances are scarce for any species and dispersal distances are not known for clouded leopards. However, a number of studies have attempted to describe allometric relationships between maximum dispersal distances and a range of physiological and life history traits for mammals in general (Bowman et al. 2002; Whitmee and Orme 2013). Bowman estimates that maximum dispersal distance is equal to 40*(home range size^0.5^) and since clouded leopards are thought to have home ranges in the region of 16km^2^ (N. diardi) - 40km^2^ (N. nebulosa) we therefore estimate that a likely range of dispersal distances from 160 -252km (Austin et al. 2007; Bowman et al. 2002; Grassman et al. 2005; Hearn et al. 2013). For this study we therefore selected 125km as a plausible conservative estimate of dispersal in clouded leopards*”. In order to test the sensitivity of our results to this estimate we also ran all analyses under an alternative dispersal scenario of 250km. This figure was selected to represent a plausible upper boundary for the maximum dispersal distance for clouded leopards. A comparison of resistant kernel maps for the 125kcu and 250kcu scenarios can be seen in Figure A2. As expected, the landscape shows higher levels of connectivity between populations under the 250kcu dispersal scenario.


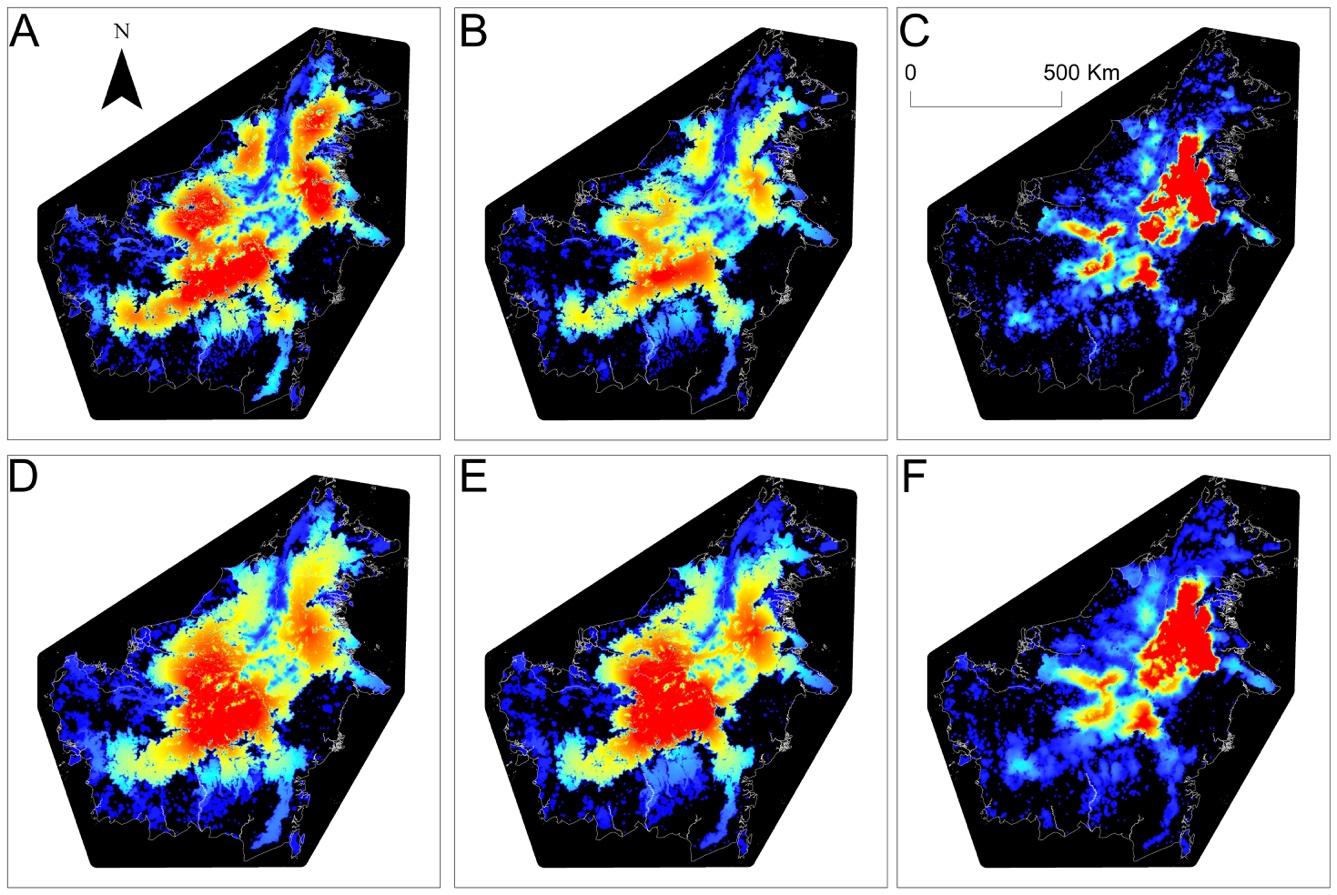


Figure A2. Comparison of resistant kernel maps for two potential dispersal distances. 125kcu (as shown in Figure 2 -,A, B, C, and 250kcu, D, E, F, for years 2000 (A and D), 2010 (B and E) and 2020 (C and F).

In addition to testing the sensitivity of our analyses to differences in dispersal distance, it was necessary for us to adopt a threshold for clouded leopard priority areas. In the main analysis we defined connected populations as those areas greater than the 10^th^ percentile of the 2000 kernel surface for all three dates. Given any particular threshold is arbitrary, we decided to explore how patterns of predicted connectivity varied across various thresholds. We therefore also tested the impact of using both the 5^th^ and 20^th^ percentile of the 2000 kernel surface as thresholds in our analysis.

All analyses were conducted at both dispersal distances and across all thresholds and the results of those analyses are presented below.


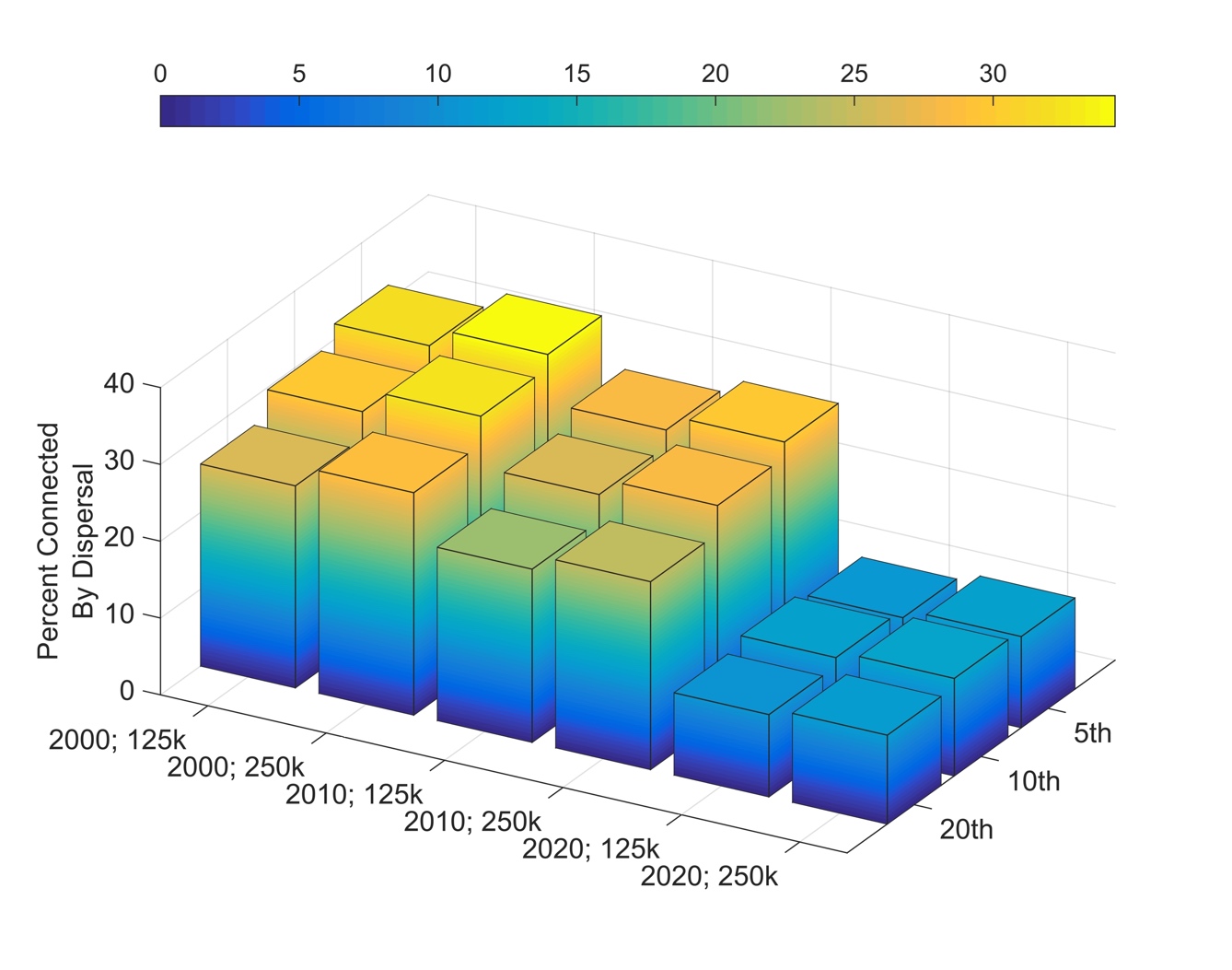


Figure A3. Change in the percentage of the landscape connected by dispersal for 2000, 2010, and 2020 across varying thresholds for selection and dispersal distance.


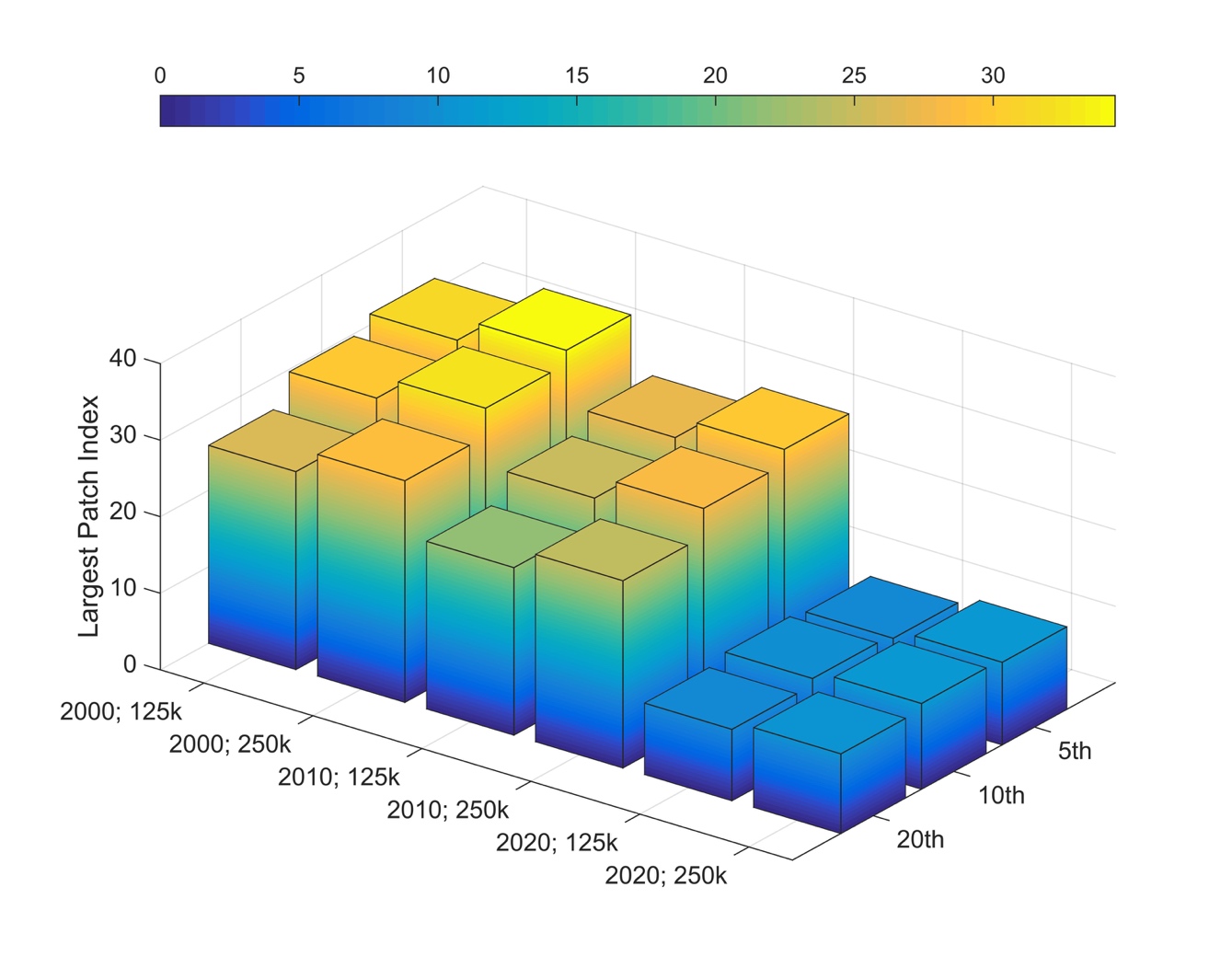


Figure A4. Change in the extent of the largest patch of connected habitat as percentage of the landscape across the 5^th^, 10^th^ and 20^th^ percentiles of the cumulative kernel surface in year 2000 for 2000, 2010, and 2020 at (a) 125kcu and (b) 250kcu dispersal thresholds.


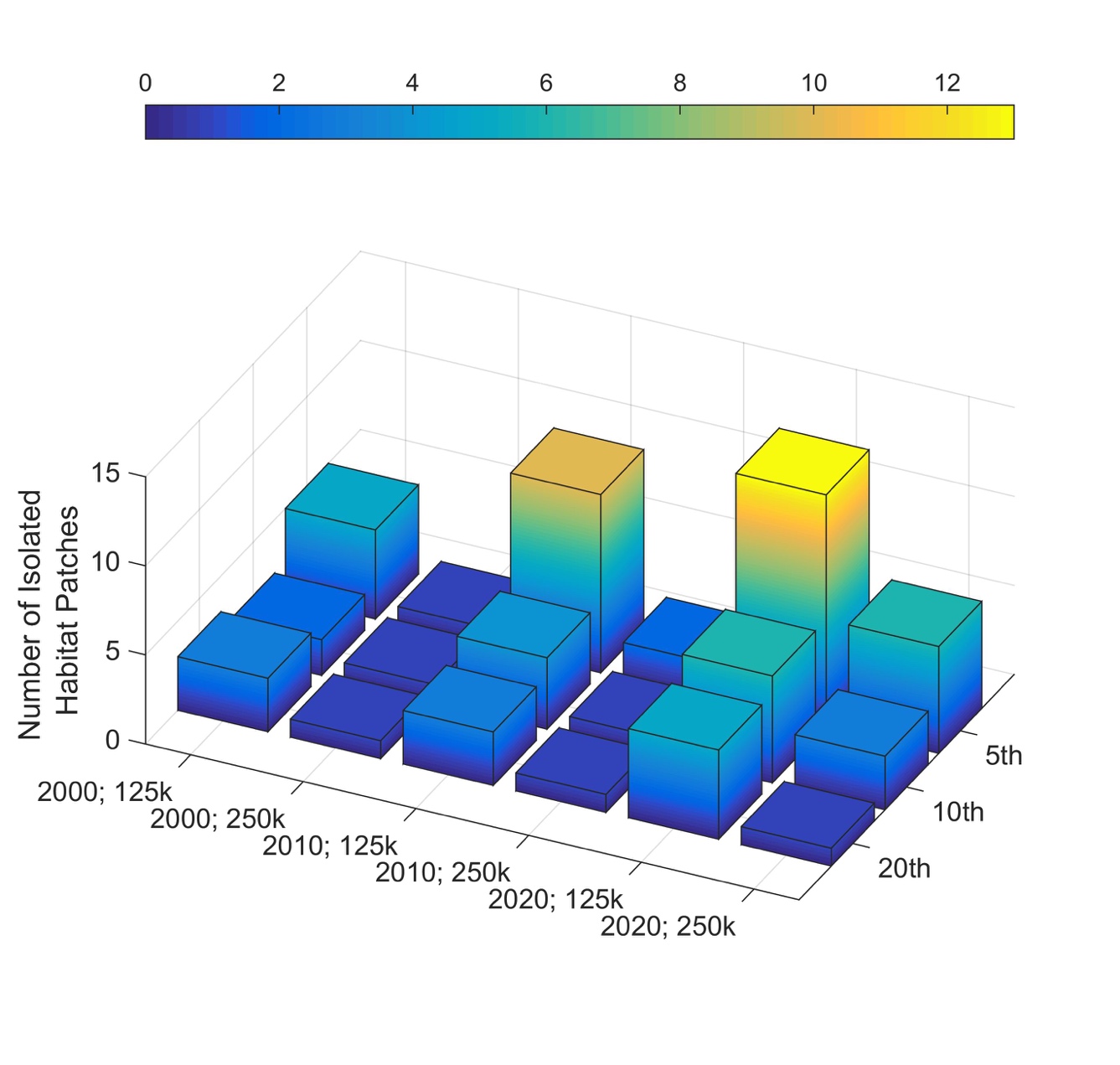


Figure A5. Change in the number of isolated patches of connected habitat across the 5^th^, 10^th^ and 20^th^ percentiles of the cumulative kernel surface in year 2000 for years 2000, 2010, and 2020 at (a) 125kcu and (b) 250kcu dispersal thresholds.


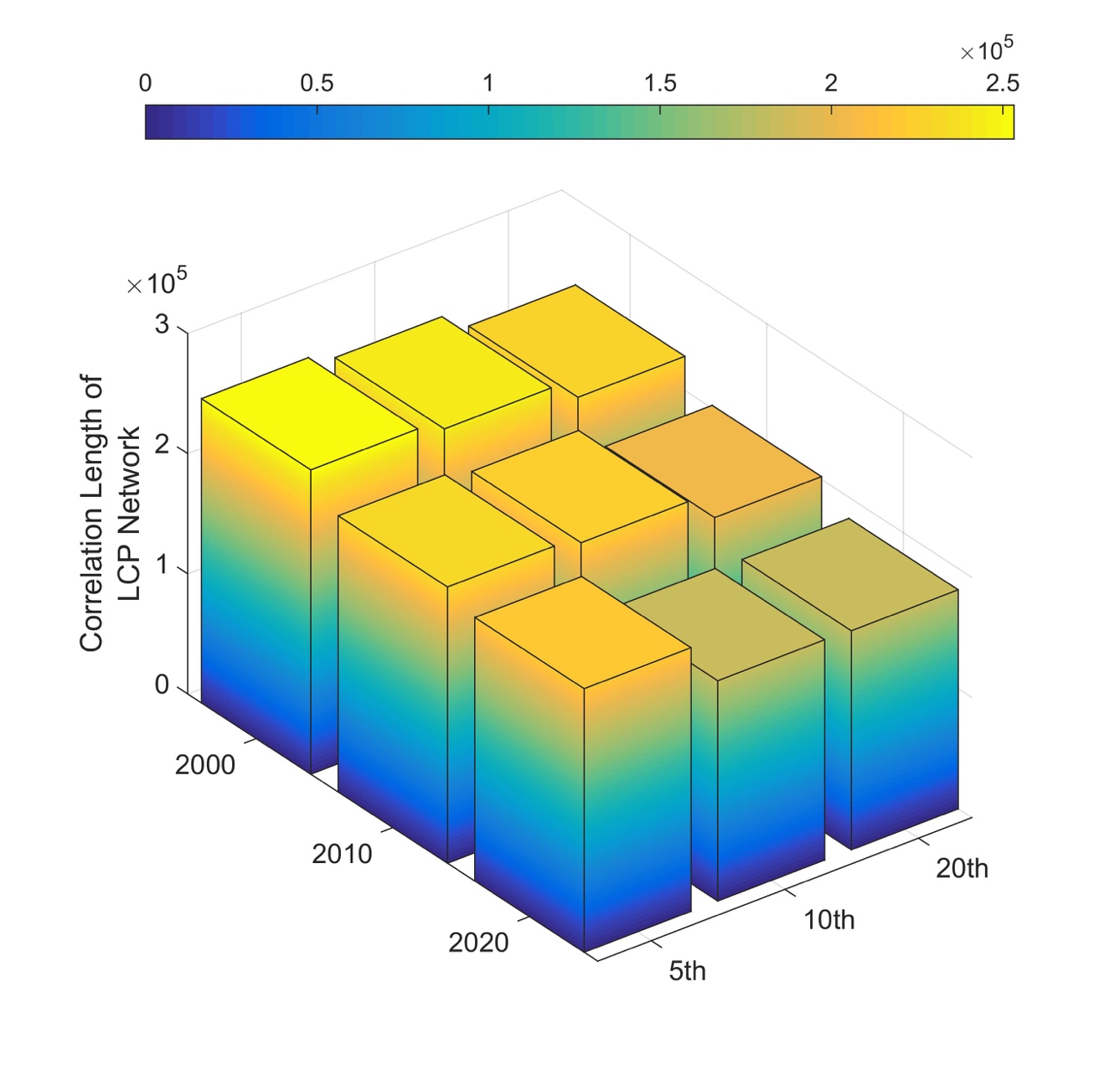


Figure A6. Changes in the correlation length of the factorial least cost path network across three density thresholds (5^th^ percentile, 10^th^ percentile and 20^th^ percentile of the least cost path density in 2000), and across the three dates (2000, 2010, 2020).

The above figures and tables show that across all landscape metrics, the impact of landscape change through time is far higher than the impact of either dispersal distance or threshold value. The only exception to this is in the number of isolated patches, which displays a complicated pattern that balances the increase in the number of patches from the fracturing of large areas against the decrease in number, as smaller patches are lost completely.

A similar pattern could be seen when we examined the impact of dispersal distance on the genetic metrics in this study. Changes through time dwarfed the effect of dispersal distance on changes in heterozygosity and local scale measures of genetic diversity.


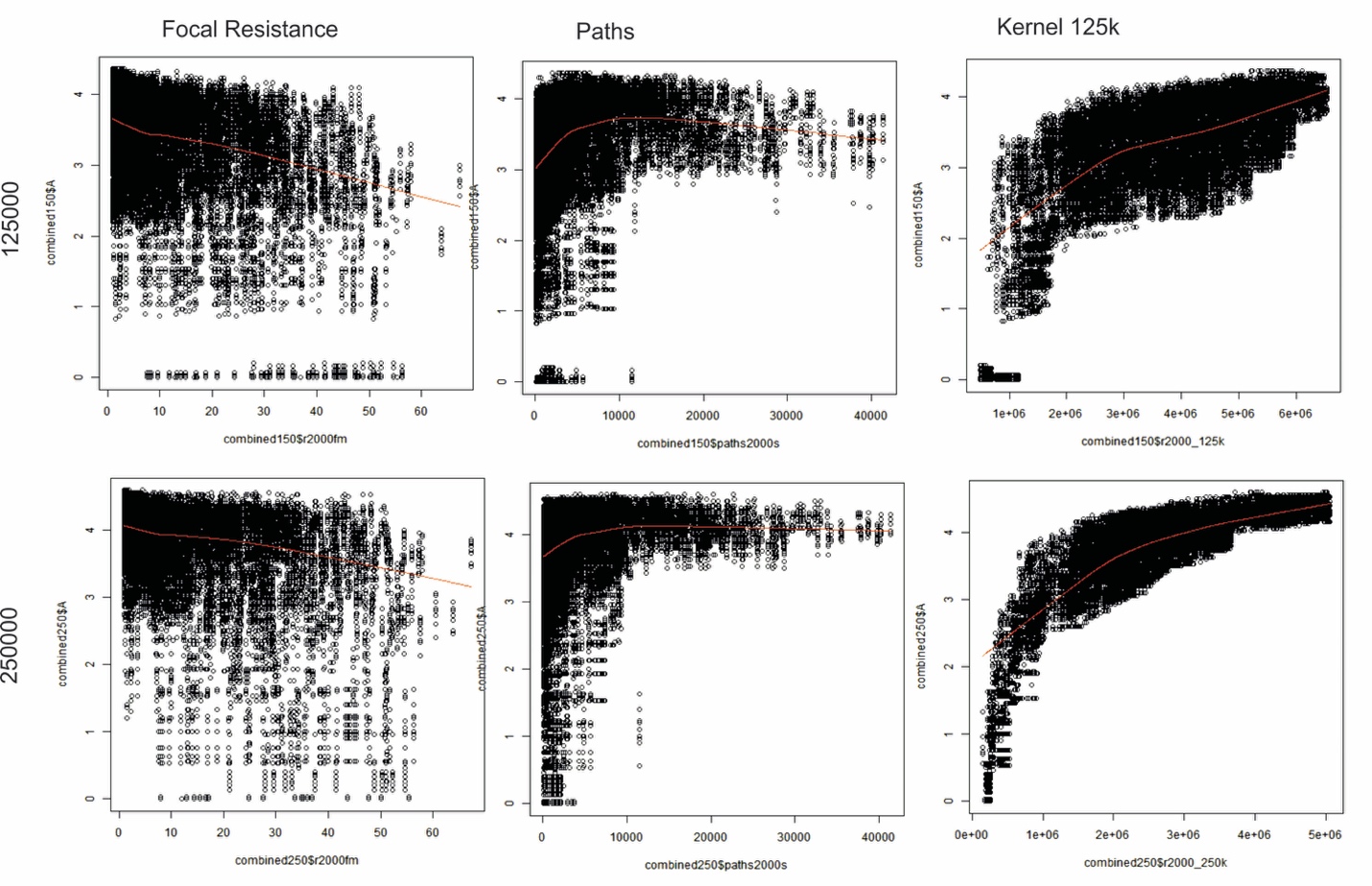


Figure A7. Scatterplots and fitted LOWESS splines for relationship between average number of alleles per locus and focal mean landscape resistance within a 10km radius (column 1), cumulative density of least cost paths (column 2), and cumulative resistant kernel density (column 3), across the two dispersal distance scenarios (125kcu, row 1; 250kcu, row 2).


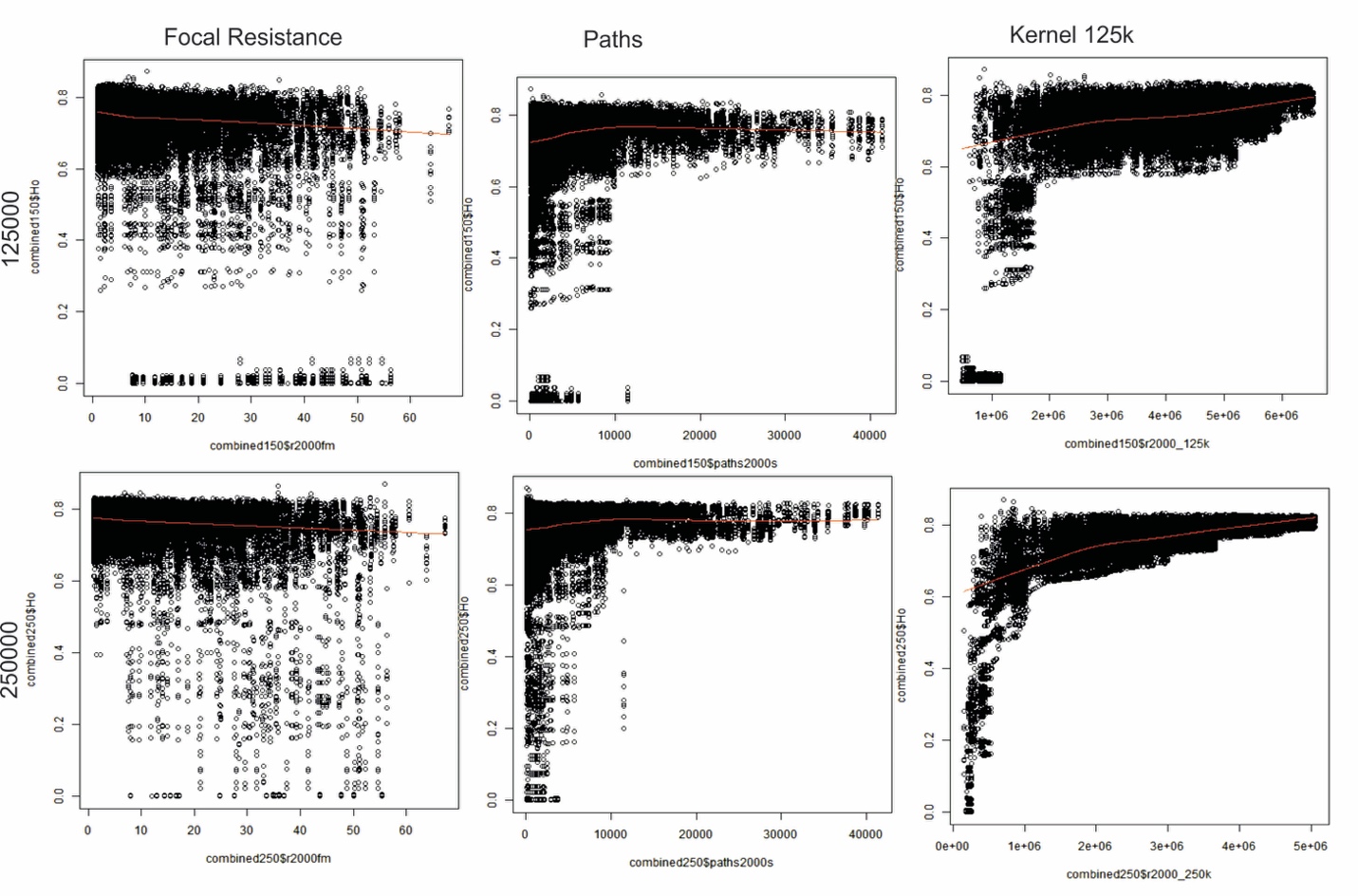


Figure A8. Scatterplots and fitted LOWESS splines for relationship between observed heterozygosity and focal mean landscape resistance within a 10km radius (column 1), cumulative density of least cost paths (column 2), and cumulative resistant kernel density (column 3), across the two dispersal distance scenarios (125kcu, row 1; 250kcu , row 2).


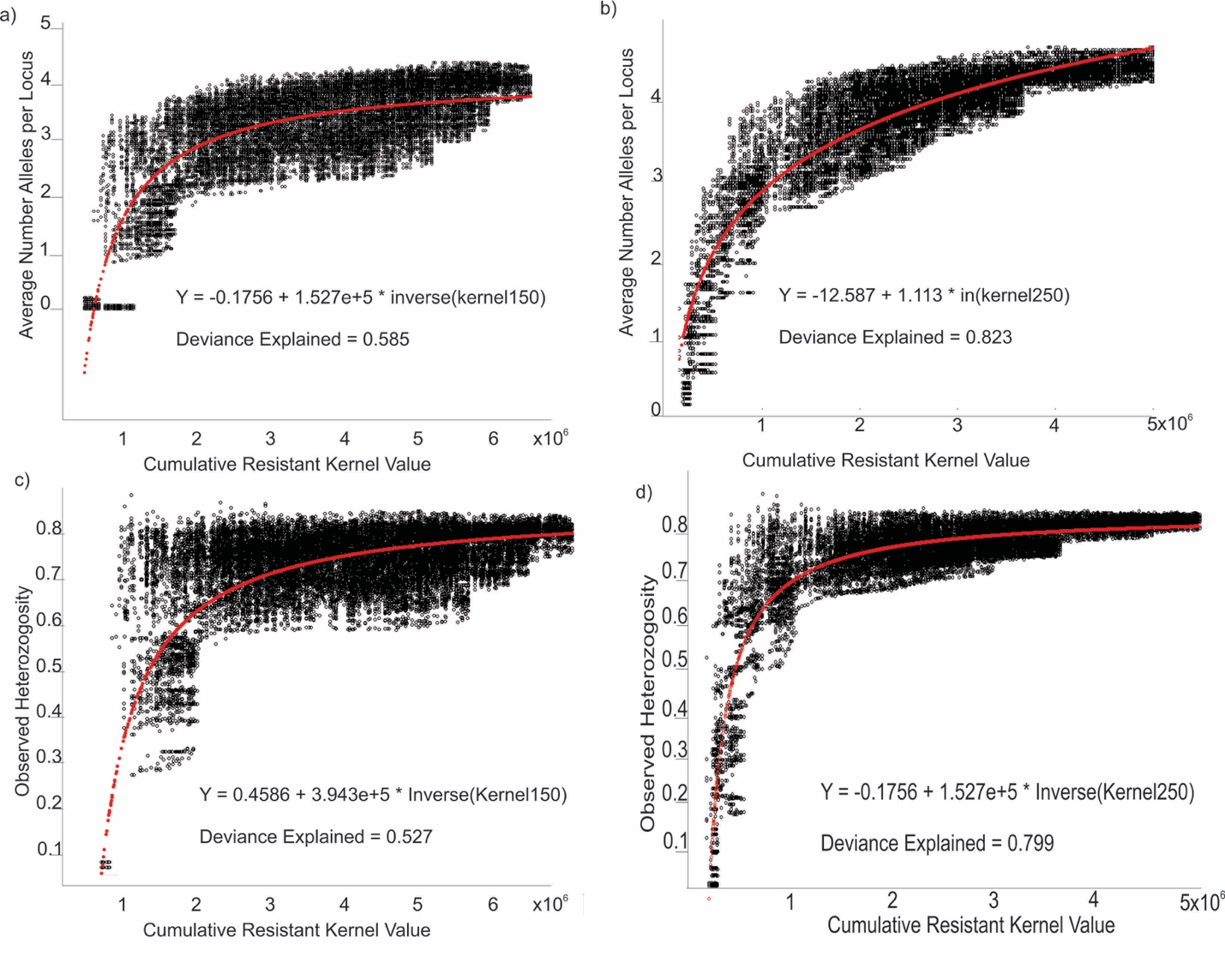


Figure A9. Scatterplots of average number of alleles per locus in a local neighborhood relative to resistant kernel value for a) the 125kcu scenario and b) the 250kcu scenario, and heterozygosity of the local neighborhood relative to resistant kernel value for c) the 125kcu scenario and d) the 250kcu scenario. The fitted regression equation is shown in red overlay, and the equation and deviance explained are displayed below each scatterplot.
